# Supplementary material for: Obstacles and facilitators of parents’ coping with hematopoietic stem cell transplantation of a child with cancer
Source: PLoS One. 2025 Oct 24;20(10):e0335013. doi: 10.1371/journal.pone.0335013 (PMC12551885; doi:10.1371/journal.pone.0335013)
Supplement: Attachment — Submitted filename: Response to Reviewers R1.docx [file pone.0335013.s004.docx]

Dear Editor, PLOS ONE

Thank you for this opportunity to revise and resubmit our article. We sincerely thank the reviewers for their comments and the provision of constructive comments to improve the quality of our article. Accordingly, text modifications were made, which have been detailed point-by-point below. All changes were highlighted throughout the manuscript using red color.

**Comments by reviewer 1**

Title and Abstract

1. The title is clear and descriptive. The abstract succinctly captures the study's purpose, methodology, key findings, and implications. However, the term “oscillating support” may be ambiguous to international readers and could benefit from rephrasing to enhance clarity (e.g., “variable support”).

**Answer:** We appreciate the reviewer’s insightful comment. To improve clarity for international readers, we have replaced the term “oscillating support” with “variable support” throughout the manuscript, including in the abstract, results, discussion, Table 2, and Figure.

Introduction

2. The introduction is informative and lays a solid groundwork for the study. The use of global and national statistics supports the relevance of the topic. The theoretical anchoring in the Transactional Model of Stress and Coping is commendable. However, the narrative could be more concise by reducing repetitive content. A clearer articulation of the research gap early in the section would also strengthen the rationale.

**Answer:** We thank the reviewer for the helpful suggestions. In response, we revised the introduction to reduce repetitive content and improve clarity while maintaining essential details. We also moved the articulation of the research gap to an earlier point in the section, emphasizing -the lack of qualitative research on parental coping in the Iranian context (Line: 47-84).

Methods

3. The methodological rigor is evident in the thorough description of the study design, ethics, and trustworthiness strategies. The use of purposive sampling and adherence to SRQR enhance credibility. Nonetheless, further details on the recruitment process, especially how participant diversity was ensured, would improve the section’s transparency. A more explicit rationale for the sample size would also be helpful.

**Answer:** To address this comment, we expanded the data collection section to provide more detail on the recruitment process and how participant diversity was ensured. Specifically, we clarified the steps taken to recruit participants from varied demographic and clinical backgrounds, including differences in gender, socioeconomic status, geographic location, and type of HSCT. We also added an explicit rationale for the sample size, explaining that the final number of 20 participants was guided by the principle of data saturation and is consistent with qualitative research standards for in-depth exploration (Line: 106-121; 132-137).

Results

4. The results are well-organized, with strong use of participant quotes to illustrate themes. The categorization into “oscillating support” and “beliefs and individual situation” effectively captures the complexity of parental coping. However, some subheadings could benefit from clearer wording. Streamlining some quotations may enhance readability without sacrificing depth.

**Answer:** We thank the reviewer for these helpful suggestions. In response, we have revised some subheadings in the Results section to improve clarity for international readers. Specifically, “Oscillating support” has been replaced with “Variable support,” and other subheadings have been reworded for greater precision (e.g., “Inadequate support from family and friends” to “Insufficient support from family and friends,” “Unfavorable support from the healthcare system” to “Challenges in support from the healthcare system,” and “Obstructive individual situation” to “Barriers related to individual circumstances”). We also streamlined several participant quotations by removing redundant details while preserving their original meaning and emotional impact, thereby enhancing readability without reducing depth.

Discussion

5. The discussion thoughtfully connects the findings to existing literature and highlights important cultural distinctions, particularly within the Iranian context. The comparison with other global studies adds value. However, there is occasional repetition of results, and the discussion could benefit from more emphasis on actionable implications. Stronger recommendations for policy or clinical practice would enhance its impact.

**Answer:** We thank the reviewer for the valuable comment. In response, we have revised the discussion to reduce repetition, clarify key findings, and emphasize actionable implications for policy and clinical practice. We highlighted the unique Iranian sociocultural and religious context in family and community support, identified systemic gaps in healthcare such as limited insurance and informal workplace support, and emphasized the critical roles of nurses and healthcare providers in facilitating communication, providing emotional and financial guidance, and supporting workplace accommodations. Additionally, we added clear recommendations, including comprehensive insurance coverage, flexible work arrangements, psychological counseling, and educational resources for parents navigating HSCT (Line: 384-513).

Limitations

6. The limitations are clearly acknowledged, including the single-center design and qualitative scope. Additional consideration of potential interviewer bias or sociopolitical influences on participant responses would provide a more comprehensive assessment.

**Answer:** We have revised the limitations section to clarify that, as a qualitative study, the findings reflect subjective experiences rather than generalizable results, and although traditional bias is not applicable, participants’ perspectives may be influenced by interviewer perspective, sociocultural or sociopolitical factors. We have highlighted the single-center and cross-sectional design limitations and suggested future multi-center and longitudinal research to enhance transferability and capture evolving coping strategies (Line: 515-526).

Conclusions

7. The conclusion succinctly summarizes key findings and emphasizes the importance of holistic support systems. It could be strengthened by offering more specific directions for future research, such as targeting particular subgroups or employing longitudinal methods.

**Answer:** We have revised the conclusion to include more specific directions for future research, highlighting the need to study particular subgroups, such as parents with limited social or financial resources, and to employ longitudinal designs to capture the evolution of coping strategies (Line: 535-538).

**Comments by reviewer 2**

This manuscript presents a timely and well-executed qualitative study that sheds light on Iranian parents’ lived experiences during their child’s haematopoietic stem cell transplantation (HSCT). The focus on both barriers and enablers of parental coping offers a balanced and nuanced contribution to the health social sciences literature. The writing is generally clear, and the integration of cultural context—particularly in the Discussion—is a commendable strength. The use of direct quotations enriches the narrative and gives voice to an often underrepresented population.

1. That said, some aspects of the manuscript would benefit from clarification and refinement. The methods section, while generally sound, needs clearer articulation of the analytical framework. Although the authors refer to conventional content analysis, their use of thematic language occasionally overlaps with approaches more typical of thematic analysis. It would strengthen the methodological rigor to either reaffirm their use of Graneheim & Lundman's content analysis or clarify if a hybrid approach was employed.

**Answer:** We appreciate the reviewer’s insightful observation. We confirm that the study employed conventional content analysis as described by Graneheim and Lundman, and not a hybrid thematic approach. To avoid ambiguity, we revised the description of the analytical process to clearly state that data were coded and categorized inductively in alignment with this method (Line: 151-160).

2. Similarly, the manuscript would benefit from more explicit engagement with issues of reflexivity—how the researchers’ positions and assumptions were acknowledged and managed during data collection and analysis.

**Answer:** We appreciate the reviewer’s comment on reflexivity. In response, we added a dedicated paragraph in the Methods section describing how the researchers’ positions and assumptions were acknowledged and managed during data collection and analysis. This includes documentation of the interviewer’s professional background, use of reflexive notes, and ongoing team discussions to mitigate bias and ensure that findings reflect participants’ perspectives (Line: 138-149).

3. Sampling procedures and inclusion criteria are generally well described; however, it would be prudent to address how gatekeeping by nurses was mitigated and to reflect on the potential bias introduced by excluding bereaved parents. Data saturation is mentioned but not operationally defined; describing how and when saturation was determined (e.g. based on codebook stability or redundancy of themes) would enhance transparency. Also, while trustworthiness criteria are cited, concrete examples illustrating credibility, dependability, and confirmability would help ground these principles in practice.

**Answer:** To address this comment, we have taken the following steps to enhance methodological clarity and transparency: (1) We clarified how gatekeeping by nurses was minimized by instructing them to introduce the study broadly to all eligible parents without pre-screening, followed by independent review of medical records by the research team (Line: 106-114); (2) We provided an operational definition of data saturation as the point when no new codes, categories, or themes emerged in three consecutive interviews and the codebook demonstrated stability (Line: 132-137); (3) We expanded the Trustworthiness section to include concrete examples of how credibility, dependability, and confirmability were achieved in practice, such as member checking, peer validation, triangulation, and maintaining an audit trail (Line: 162-176); (4) In the Limitations section, we acknowledged the exclusion of bereaved parents as a potential source of bias that may have limited the diversity of coping experiences captured in the study (Line: 515-526).

4. The results section presents insightful findings, though the category structure could be refined. Some sub-categories conflate sources of support with the adequacy of that support, which may obscure the analytic clarity. The Discussion would benefit from reducing repetition of verbatim results and instead focusing on deeper interpretive synthesis. Additionally, the authors could specify which systems or actors might deliver the “comprehensive support” they recommend, to strengthen the paper’s applicability.

**Answer:** We appreciate the reviewer’s insightful comments on the Results section. In revising the manuscript, we refined the wording of certain category and subcategory titles to improve clarity and ensure that their focus is easily understood. We also streamlined some of the participant quotations by removing non-essential details while preserving their original meaning, thus enhancing readability without losing depth. While we acknowledge the potential to further separate “sources of support” from “adequacy of support”, we decided to retain the overall category structure because it reflects the way participants themselves intertwined these aspects in their narratives. Many participants described the source and adequacy of support in a single account, making it analytically meaningful to present them together. We believe that maintaining this integrated structure preserves the authenticity of participants’ experiences and remains consistent with the inductive nature of our conventional content analysis (Result section).

In the Discussion, we have reduced repetition of verbatim participant quotations and placed more emphasis on interpretive synthesis, linking our findings to existing literature and theoretical frameworks. Additionally, as recommended, we have added a concluding paragraph specifying which systems and actors—such as multidisciplinary HSCT teams, hospital-based counseling services, non-governmental organizations, and community support networks—could deliver the comprehensive support identified as necessary in our study (Line: 505-513).

5. Finally, a few editorial adjustments—such as consistent tense usage, use of definite articles (e.g. “the largest Children’s Medical Center”), and spelling out abbreviations—would improve readability.

**Answer:** We thank the reviewer for the editorial suggestions. We have carefully standardized tense usage, ensured correct use of definite articles, and spelled out all abbreviations at their first mention throughout the manuscript to improve clarity and readability.

**Comments by reviewer 3**

With the exception of replacing "1th" with "1st" and "2th" with "2nd", [Table 1], no changes are recommended. There is an orderly presentation of the results, which is a nicely detailed listing of important findings; the alternating description-quote for this evidence writing style is actually nicely 'to the point.' I might have expected a short listing or two interspersed here--but that probably doesn't add anything essential, and numbers of words limits to publications/length of article allow us to ignore that point. The value of applying qualitative research methodology for exploratory purposes, involving new therapeutics, is well illustrated here. The placement of barriers to change (patient/self, family/friends, associates/professionals, larger parts of society/culture) are well demonstrated here, and easy to review and make use of. There are certain underlying sociocultural aspects of this research subject and approach that make this article importantly definitive of the interculturalism, internationally shared qualities of life, aspects of health, and needs regarding health care, especially for conditions that are equally important across all disciplines and people. Sometimes, we relate quality of life and health care to the kind of society or country health care is taking place within, with the added impression that quality of care can differ greatly across different cultures and countries. But this article clearly demonstrates that certain human features about QOL and health care, that exist regarding of a culture or country's place in the world. This article puts that concept back into the conscious mindset of its readers, and other nations' healthcare givers. It reminds us that regardless of quality of care, availability of care, cost of care, most health matters are global, and most nations, cultures, need to be acknowledged for the efforts being made in handling their highest risk patients. This article is a very nice, detailed yet succinct example that I would share with my students, in my qualitative research methods course teachings.

**Answer:** We sincerely thank the reviewer for the thoughtful and encouraging feedback. We greatly appreciate your recognition of the clarity, organization, and level of detail in our results presentation, as well as your acknowledgment of the value of applying qualitative methodology to this research topic. We are also grateful for your insightful reflections on the intercultural and globally relevant aspects of quality of life and health care illustrated in our findings.

As suggested, we have corrected “1th” to “1st” and “2th” to “2nd” in Table 1. We are encouraged by your remarks that the manuscript provides a succinct yet detailed example of qualitative research that could be useful in teaching contexts. Your positive evaluation reinforces the importance of the study’s contribution and motivates us to further pursue research on this topic.

**Comments by reviewer 4**

You have taken on an important and emotionally complex topic by exploring the experiences of parents whose children undergo hematopoietic stem cell transplantation. I understand the inherent difficulties of conducting in-depth qualitative research with this vulnerable population, and I appreciate your effort. I provide several helpful recommendations below to improve your manuscript's theoretical alignment, methodological clarity, and transparency.

1. Reflexivity is not adequately addressed in the manuscript. Important details about the research team are absent, such as: Which author or authors conducted the interviews? What qualifications and qualitative methods training did they have? Which gender were they? Before the interviews, were relationships built with the participants? This information is required to assess possible influences on data collection and interpretation. In the Methods section, I suggest adding a succinct paragraph describing the interviewers' backgrounds and any connections to participants.

**Answer:** We thank the reviewer for noting the need for more detail on reflexivity and the research team’s background. We have now specified which author conducted the interviews, their gender, qualifications, qualitative research training, and clinical experience with HSCT. We also clarified that no prior personal relationships existed with participants, that rapport was built through initial contact and informed consent, and that reflexive strategies were applied to minimize potential influence on data collection and interpretation (Line: 138-149).

2. Several important study design elements are not disclosed: How participants were approached (e.g., face-to-face, telephone, etc.) is unclear. There was no mention of whether any individuals refused to participate or dropped out or whether refusals were treated as exclusion criteria. It is also not stated whether field notes were made during or after the interviews.

**Answer:** We have now clarified that participants were approached either face-to-face during hospital visits or by telephone using contact details from medical records, with prior ethics approval. We have also specified that five bereaved parents were excluded to avoid exacerbating grief, eight eligible parents declined to participate, and four was excluded because their child was undergoing HSCT at the time. In addition, we have indicated that field notes were made during and after each interview to capture contextual observations, non-verbal cues, and the interviewer’s reflections (Line: 106-121; 132-137).

3 The description of the data analysis process lacks several critical details: How many researchers were involved in coding the data? What software was used to manage the data? Were the themes derived inductively or identified in advance? Did participants review the findings (member checking)? How were discrepancies between coders resolved? How did you use triangulation strategies to investigate data validity?

**Answer:** We thank the reviewer for highlighting the need for additional detail in the data analysis section. We have now specified that two researchers independently conducted the coding manually in Microsoft Word, without specialized software, and that discrepancies were resolved through discussion or, if necessary, by consulting a third author. We also clarified that themes and categories were derived inductively from the data. Details regarding member checking and triangulation are addressed in the Trustworthiness section (Line: 151-176).

4. Despite claiming in the introduction that the study is grounded inn Lazarus and Folkman's Transactional Model, the methodology, analysis, or discussion do not significantly incorporate the model. The interview guide, coding framework, or thematic analysis have not been organized according to the model's central concepts (primary and secondary appraisal, for example). I suggest specifically integrating the model into the analytical and discussion sections to guarantee theoretical consistency.

**Answer:** We thank the reviewer for this observation. As our study employed conventional content analysis, categories were derived inductively from the data rather than being pre-structured according to the Transactional Model. This methodological choice ensured that findings emerged directly from participants’ narratives. Nevertheless, to strengthen theoretical consistency, we have now integrated an explicit interpretation in the Discussion section linking our inductively derived categories to the key concepts of the model, particularly primary and secondary appraisal (Line: 496-504).

We hope that the changes meet your journal needs.

Sincerely Yours,

*Authors*
