# Supplementary material for: Obstacles and facilitators of parents’ coping with hematopoietic stem cell transplantation of a child with cancer
Source: PLoS One. 2025 Oct 24;20(10):e0335013. doi: 10.1371/journal.pone.0335013 (PMC12551885; doi:10.1371/journal.pone.0335013)
Supplement: Attachment — Submitted filename: Comments for 19344 R1.docx [file pone.0335013.s003.docx]

Based on my re-review of the manuscript, "Obstacles and facilitators of parents' coping with hematopoietic stem cell transplantation of a child with cancer," I have concluded that the authors have successfully addressed the concerns raised by the reviewers. The revisions have significantly improved the manuscript, and it is now suitable for publication.

Here is a summary of the key revisions and how they have improved the manuscript:

- **Clarity and Readability**: The authors have revised the title and abstract, replacing the ambiguous term "oscillating support" with "variable support" to improve clarity for international readers. They also reworded several subheadings in the results section for greater precision.
- **Methodological Rigor and Transparency**: The authors have provided a more explicit rationale for their sample size, explaining that the final number of 20 participants was guided by data saturation. They also clarified the recruitment process to ensure participant diversity. Furthermore, they added a dedicated paragraph on reflexivity, detailing how the researchers' positions and assumptions were managed during data collection and analysis. The manuscript also now includes an operational definition of data saturation and concrete examples of how trustworthiness criteria were met, such as member checking and peer validation.
- **Theoretical Consistency**: While the study used a conventional content analysis method, the authors have now integrated an explicit interpretation in the discussion section to link their inductively derived categories to the key concepts of the Transactional Model of Stress and Coping, strengthening the paper's theoretical consistency.
- **Actionable Implications**: The discussion has been revised to reduce repetition and place more emphasis on actionable implications for policy and clinical practice. The authors added clear recommendations for comprehensive support systems, including roles for multidisciplinary teams, hospital-based counseling, and community networks.
- **Limitations and Bias**: The authors have acknowledged the potential bias introduced by excluding bereaved parents from the study and noted that the findings are specific to a single institution in Iran, limiting their generalizability. They also added a note on potential influences from the interviewer and socio-political factors.

The authors' thorough and well-reasoned responses demonstrate their commitment to improving the manuscript. The revisions have not only addressed the specific points raised by the reviewers but have also strengthened the overall quality and contribution of the paper.
