# Supplementary material for: Obstacles and facilitators of parents’ coping with hematopoietic stem cell transplantation of a child with cancer
Source: PLoS One. 2025 Oct 24;20(10):e0335013. doi: 10.1371/journal.pone.0335013 (PMC12551885; doi:10.1371/journal.pone.0335013)
Supplement: Attachment — Submitted filename: Response to Reviewers R2.docx [file pone.0335013.s005.docx]

Dear Editor, PLOS ONE

Thank you for this opportunity to revise and resubmit our article. We would like to express our sincere gratitude to all the reviewers for their thoughtful feedback. We especially thank the two reviewers who endorsed our manuscript for publication, as well as the reviewer who provided detailed suggestions for revision. Their constructive comments were invaluable in improving the clarity, theoretical integration, and overall quality of our work.

Accordingly, some text modifications were made and are presented point-by-point below. All changes have been highlighted throughout the manuscript using red color.

*Sincerely Yours*

**Comments by reviewer 2**

I appreciate the authors' response and the integration of the Transactional Model of Stress and Coping into the Discussion section, as this adequately addresses my previous comment. However, it would have been beneficial if the authors had demonstrated more explicitly how the concepts of primary and secondary appraisal influenced the coding or theme generation. Since these concepts are central to the theoretical framework, further clarification of their role in shaping the analysis would strengthen the theoretical consistency and transparency of the study's methodology.

**Answer:** Thank you very much for your constructive feedback. We would like to clarify that our study employed a conventional content analysis approach, rather than a directed content analysis. In conventional content analysis, categories are inductively generated from the data without being constrained by a predefined theoretical framework. Therefore, the coding and theme development in our study were grounded in participants’ narratives rather than explicitly guided by the concepts of primary and secondary appraisal.

However, in line with your valuable suggestion, we sought to connect our findings to the Transactional Model of Stress and Coping at the stage of interpretation and discussion. By doing so, we aimed to demonstrate how the parents’ experiences—such as fear, uncertainty, financial strain, and reliance on family and spiritual support—can be understood through the processes of primary and secondary appraisal. This approach allowed us to preserve the inductive integrity of conventional content analysis, while still situating our findings within a meaningful theoretical framework (Line: 381-388; 427-430; 466-469; 493-495).

**Comments by reviewer 4**

Thank you for the response. However, I was unable to locate the claimed integration of the Transactional Model in the Discussion section. The manuscript still lacks a meaningful theoretical framework that connects findings to broader analytical concepts. Without this, the study remains descriptive and does not move beyond context-specific observations. Theory provides the necessary link between observing something and giving it meaning.

**Answer:** We sincerely thank you for your valuable comment. In response, we have revised the Discussion section to more explicitly integrate the Transactional Model of Stress and Coping as the theoretical framework. Specifically, we now interpret our findings in terms of primary appraisal (e.g., parents’ reported fear, financial stress, and conflict with healthcare teams, which reflect the perception of HSCT as a highly stressful and threatening situation) and secondary appraisal (e.g., parents’ references to spousal support, NGO assistance, financial stability, and spiritual beliefs, which represent resources that helped them to cope). We further highlighted that when resources were perceived as sufficient, parents described resilience and hope, while lack of resources undermined coping and intensified stress (Line: 381-388; 427-430; 466-469; 493-495).

We hope that the changes meet your journal needs.

Sincerely Yours,

*Authors*
